# The effect of a dopamine antagonist on conditioning of sexual arousal in women

**DOI:** 10.1007/s00213-015-4201-x

**Published:** 2016-02-02

**Authors:** Mirte Brom, Ellen Laan, Walter Everaerd, Philip Spinhoven, Baptist Trimbos, Stephanie Both

**Affiliations:** Institute of Psychology, Clinical Psychology Unit, Leiden University, Wassenaarseweg 52, Leiden, 2333 AK The Netherlands; Department of Sexology and Psychosomatic Obstetrics and Gynaecology, Academic Medical Centre, University of Amsterdam, Meibergdreef 9, 1105 AZ Amsterdam, The Netherlands; Department Clinical Psychology, University of Amsterdam, Weesperplein 4, 1018 XA Amsterdam, The Netherlands; Department of Psychiatry, Leiden University Medical Centre, P.O. Box 9600, 2300 RC Leiden, The Netherlands; Department of Gynaecology, Leiden University Medical Centre, P.O. Box 9600, 2300 RC Leiden, The Netherlands; Department of Psychosomatic Gynaecology & Sexology, VRSP, Leiden University Medical Centre, Rijnsburgerweg 10, Zone PG4-Z, P.O. Box 9600, 2300 RC Leiden, The Netherlands

**Keywords:** Sexual conditioning, Dopamine antagonist, Sexual reward learning, Sexual response, Haloperidol

## Abstract

**Rationale:**

Dopamine (DA) plays a key role in reward-seeking behaviours. Accumulating evidence from animal and human studies suggests that human sexual reward learning may also depend on DA transmission. However, research on the role of DA in human sexual reward learning is completely lacking.

**Objectives:**

To investigate whether DA antagonism attenuates classical conditioning of sexual response in humans.

**Methods:**

Healthy women were randomly allocated to one of two treatment conditions: haloperidol (*n* = 29) or placebo (*n* = 29). A differential conditioning paradigm was applied with genital vibrostimulation as unconditional stimulus (US) and neutral pictures as conditional stimuli (CSs). Genital arousal was assessed, and ratings of affective value and subjective sexual arousal were obtained.

**Results:**

Haloperidol administration affected unconditional genital responding. However, no significant effects of medication were found for conditioned responding.

**Conclusions:**

No firm conclusions can be drawn about whether female sexual reward learning implicates DA transmission since the results do not lend themselves to unambiguous interpretation.

## Introduction

The dopaminergic reward system has been implicated to be involved in the acquisition and expression of learned appetitive behaviours (Dominguez and Hull 2005; Fields et al. 2007; Richard et al. 2013; Schultz 2002), and abnormality in this system has been shown to play a role in the aetiology and pathophysiology of various disorders, including substance use disorders and (behavioural) addictions (De Jong et al. 2015; Dunlop and Nemeroff 2007; Root et al. 2015). Many theories of human sexual behaviour assume that sexual stimuli can obtain arousing properties through associative (classical/Pavlovian) learning processes (Brom et al. 2014a; Pfaus et al. 2001; Toates 2009). Therefore, the onset of disorders in sexual motivation such as female sexual interest/arousal disorder (Diagnostic and Statistical Manual of Mental Disorders [DSM-5] American Psychiatric Association 2013) or hypersexuality may be explained from a classical conditioning and incentive motivation perspective (Brom et al. 2014a; Laan and Both 2008; Singer and Toates 1987). However, despite the substantial amount of research that suggests that mesolimbic dopamine (DA) neurotransmission plays an important role in aversive learning (Zweifel et al. 2011), as well as reward learning (Berridge 2007; Berridge and Robinson 1998, 2003; Brom et al. 2014a; Di Chiara 1995; Kringelbach and Berridge 2009), to date, no human research has been conducted on the role of DA in human sexual reward learning, while facilitation as well as impairment thereof is relevant in the context of treatment of sexual motivation disorders.

Stimuli that can promote motivation are called incentive stimuli (Bindra 1974; Singer and Toates 1987). Their motivational valence can be unconditional or conditional as a result of associative leaning (Di Chiara 1995). A previously neutral stimulus (NS) that predicts reward (i.e. unconditional stimulus (US)) can acquire motivational properties, becoming an attractive and desirable incentive stimulus (i.e. conditional stimulus (CS)). As a result of repeated association of the NS with the US, the NS may eventually trigger similar responses as the US (i.e. the conditioned response (CR)) (Pavlov 1927). However, it is important to mention that the NS does not always have to trigger the exact same response as the US does (Fanselow et al. 1994), and therefore, the CR may not always equal the unconditioned response (UR). Subsequent repeated presentations of a CS without the US will result in a loss of conditioned responding (i.e. extinction), as the CS no longer predicts the appetitive US (Delamater 2004). Several studies have demonstrated conditioned sexual arousal responses in animals and humans (Both et al. 2011; Brom et al. 2014a, b, c; Pfaus et al. 2001).

Rewards like food, drugs and sex have the ability to stimulate mesolimbic DA neurons projecting from the ventral tegmental area (VTA) to the nucleus accumbens (NAc), and increased extracellular concentrations of mesolimbic DA are implicated in responding for conditioned reinforcers (Berridge 2007; Georgiadis and Kringelbach 2012; Pierce and Kumaresan 2006; Richard et al. 2013). A recent functional MRI (fMRI) study (Oei et al. 2012) provides compelling evidence for a mediating role of DA in processing of subconscious perceived sexual stimuli. In healthy young men, levodopa (a DA agonist) enhanced the activation in the NAc and dorsal anterior cingulate cortex in response to subliminal sexual stimuli, whereas haloperidol decreased activations in those areas. Both et al. (2005) demonstrated a relation between dopaminergic activity and motor preparation in response to sexual stimuli. Moreover, substantial evidence suggests that mesolimbic DA plays a critical role in the incentive and acquisition aspect of reward (Berridge 2007; Schultz 2002; Wise 2002). The incentive salience theory describes mechanisms by which DA transmission in the NAc transforms the neural representations of conditioned stimuli, converting an event or environmental stimulus from a neutral representation into an attractive and ‘wanted’ incentive (Berridge 2007; Flagel et al. 2010). Research has shown that DA agonists or DA uptake inhibitors, such as D-amphetamine or methylphenidate, increase conditioned responding in rats (Beninger and Phillips 1980; Cummins et al. 2014; Taylor and Robbins 1984) and humans (Kassubek et al. 2011), whereas DA antagonists decrease conditioned responding in rats (Banasikowski et al. 2010; Ranaldi and Beninger 1993; Wolterink et al. 1993). For instance, in rats, haloperidol selectively attenuates conditioned cue-induced sexual motivation (Coria-Avila et al. 2008; López and Ettenberg 2002). In humans, dopaminergic influences on reward learning were observed in studies by Pessiglione et al. (2006) and Pleger et al. (2009), in which participants were administered a single dose of haloperidol or levodopa preceding an instrumental learning task and a reward decision-making task respectively. Haloperidol attenuated, and levodopa enhanced learning effects. However, in contrast, Pizzagalli et al. (2008) and Santesso et al. (2011) demonstrated that a single dose of the DA agonist pramipexole impaired the acquisition of reward-related behaviour in healthy participants. This blunted reward learning was explained by the assumption that low doses of pramipexole may influence reward via a paradoxical effect related to activation of the presynaptic DA autoreceptor, resulting in a blockade of phasic DA release and a blunted response to rewarding stimuli (Riba et al. 2008). As the mixed results make clear, the role of DA signalling in incentive learning in humans remains largely unknown. Therefore, in the present study, making use of a double-blind, placebo-controlled design, it was investigated whether DA antagonism attenuates classical conditioning of sexual response in women. It was expected that administration of the DA antagonist haloperidol would decrease the magnitude of the conditioned sexual response.

## Method

### Participants

A total of 58 healthy sexually active women from the general population were recruited by means of advertisements and were randomly allocated to two treatment conditions: placebo *n* = 29 and haloperidol *n* = 29. The inclusion criteria were age between 18 and 45 years and a heterosexual orientation; no pregnancy or breastfeeding; no current (or history of) sexual complaints as determined by the Female Sexual Function Index (FSFI; Rosen et al. 2000; Ter Kuile et al. 2006) or psychiatric problems as determined by the MINI International Neuropsychiatric Interview (MINI; Sheehan et al. 1998); no history of sexual abuse; no medical illness (or medical history) indicating a risk in using haloperidol (e.g. cardiac illness, depression, thyroid disorders, glaucoma); no use of medication affecting sexual response and no current or recent use (<12 weeks before participation) of psycho-pharmacological medication, psychotropic drugs or medication that might interfere with haloperidol (e.g. cannabis or cocaine). Participants were paid €50 for their participation, and written informed consent was obtained from all participants. The study was approved by the Medical Ethics Committee and carried out according to the standards of the Declaration of Helsinki (Declaration of Helsinki 2000).

### Medication

Participants received a single dose of haloperidol (3 mg, the mean time of maximal plasma levels (*T*_max_) = 3–6 h, half-time = 14–36 h; Liem-Moolenaar et al. 2010) or placebo (microcristalline cellulose), hidden in identical gelatine capsules to ensure that both participants and experimenters could not identify the drugs. Following dosing, participants rested for 3 h to allow drug absorption. This timing was based upon a studies in healthy volunteers that showed 60–70 % D2 receptor occupancy and maximal plasma concentrations 3 h after haloperidol administration (Darby et al. 1995; Nordstrom et al. 1992) and on research that showed haloperidol effects 1 h after dosing on cognitive tests making use of a reliable central nervous system (CNS) measurement battery (Liem-Moolenaar et al. 2010). Moreover, previous studies from our lab (Oei et al. 2012) demonstrated decreased activations in brain reward structures 4 h after oral ingestion of 3 mg haloperidol.

### Conditioning procedure

**Fig. 1:**
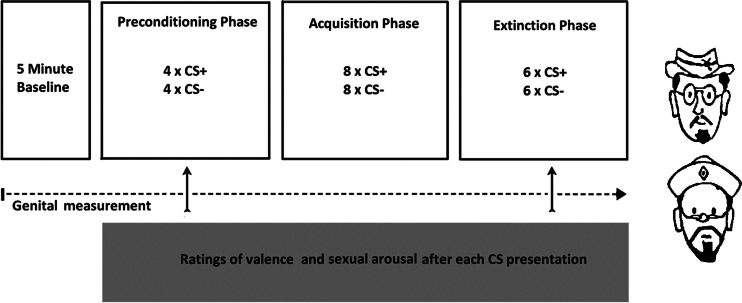
Schematic representation of the conditioning procedure and extinction phase, with on the *right* the used stimuli that served as CSs

The experimental design involved differential conditioning with one stimulus (the CS+) being followed by genital vibrostimulation (US) during the acquisition phase, whereas the other stimulus (CS−) was never followed by genital vibrostimulation. For a schematic overview of the procedure, see Fig. 1. In the preconditioning phase, participants saw four non-reinforced presentations of the CS+ and four presentations of the CS−, for 11 s each. Subsequently, in the acquisition phase, the CS+ and CS− were presented eight times each, and after 10 s, the CS+ was always followed by the US for 2 s. In the extinction phase, the CS+ and CS− were presented six times each, and now, the CS+ was no longer followed by the US. All phases were presented without interruption. There were two random CS orders for each phase (that was counterbalanced across participants), with the restriction of only two successive presentations of each CS. During the whole procedure, inter-trial intervals (ITIs) were 20, 25 or 30 s. The order of the length of the ITI was random, with the restriction of only two similar successive lengths.

### Stimulus materials

Two similar neutral pictures of pictorial male faces (Both et al. 2011; Brom et al. 2014b) served as CSs. The CSs were shown in the middle of a computer monitor, approximately 1.5 m in front of the participant. The size of the presented pictures was 14 × 21 cm. Assignment of the pictures as CS+ and CS− was counterbalanced across participants and conditions. A computer program timed the administration of the CS and US stimuli.

### Genital vibrostimulation (US)

A small hands-off vibrator (2-cm diameter) (Both et al. 2008, 2011; Brom et al. 2014b) was placed on the clitoris using a lycra panties. All participants were instructed to position the vibrator as *most sexually stimulating*.

### Genital arousal

Vaginal photoplethysmography assessed vaginal pulse amplitude (VPA) (Laan and Everaerd 1995; Laan et al. 1995). VPA is a reliable measure specific to sexual arousal (Laan et al. 1995; Suschinsky et al. 2009). The photoplethysmograph is a menstrual tampon-sized device containing an orange-red light source and a photocell. The light source illuminates the capillary bed of the vaginal wall and the blood circulation within it. Depth of the probe and orientation of the light emitting diode were controlled by a device (a 6 × 2-cm plate) attached to the cable within 5 cm of the light sensor. The photoplethysmograph was disinfected at the medical centre by means of a plasma sterilization procedure between uses. Plasma sterilization is a highly effective method for the complete removal of all organic (and certain inorganic) material. Genital response was measured continuously during resting baseline, preconditioning, acquisition and extinction phases.

### Subjective ratings

Ratings of affective value and sexual arousal were collected during the preconditioning and extinction phases. Participants were first asked to rate, after each CS presentation, the affective value of the CSs by answering the question ‘*What kind of feeling does this picture evoke in you*?’ The question could be answered on a 7-point Likert scale on a keyboard that varied from *very negative* to *very positive*. Then, subjective sexual arousal was rated by answering the question ‘*How sexually arousing is this picture to you*?’ The question could be answered on a 7-point scale that varied from *not sexually arousing at all* to *very sexually arousing*. The questions were presented at the monitor 1 s following the end of picture presentation.

### The Female Sexual Function Index

Women’s sexual functioning was assessed by the FSFI (Rosen et al. 2000; Ter Kuile et al. 2006), consisting of six subscales: desire (two items; range 1–5), arousal (four items; range 0–5), lubrication (four items; range 0–5), orgasm (three items; range 0–5), satisfaction (three items; range 0–5) and pain (three items; range 0–5). A higher score indicates better sexual functioning. The FSFI has good internal reliability and is able to differentiate between clinical samples and functional controls (Rosen et al. 2000; Ter Kuile et al. 2006).

### Procedure

A female who was a trained experimenter tested each participant individually. Women were not tested during menstruation. Before entering the experimental session, participants were instructed about the genital device and vibrostimulation and informed consent was obtained. Participants received a capsule (placebo or haloperidol) 3 h before the experimental conditioning procedure to ensure the occurrence of peak plasma concentrations of the drugs during the experiment (Darby et al. 1995; Liem-Moolenaar et al. 2010; Nordstrom et al. 1992). After ingestion of the capsule, participants filled out questionnaires (e.g. FSFI). They were allowed to read during the waiting period. Exactly 3 h after ingesting the capsule, the experimental procedure started. Participants privately inserted the vaginal device and placed the vibrator. Further instructions were given through instructions on screen. Three presentations of vibrostimulation of 2 s each allowed the participant to position the vibrator in the way it was *most sexually arousing*. It was emphasized that after final placement, the position of the vibrator should not be changed during the experiment. A 5-min resting period followed, during which a neutral film was played and baseline measurements of genital response were collected during the last 2 min. Subsequently, the conditioning procedure followed. After the experiment finished, participants themselves removed the genital devices privately. Next, a debriefing interview was administered in which participants were asked about their sentiments with regard to the experimental procedure, the use of the genital device and their evaluation of the genital vibrostimulation. Finally, participants were thanked and paid for their participation and advised to refrain from alcohol and drug use the next 24 h.

### Data reduction, scoring and analysis

A software program (VSRRP98) was used to analyze the genital data. After artefact removal, mean VPA level during the 2-min resting baseline period was calculated. Genital responses to the CSs were scored in three latency windows: during 4–8, 9–12 and 13–16 s following CS onset, respectively first interval response (FIR), second interval response (SIR) and third interval response (TIR). These time intervals are based on previous data (Both et al. 2011; Brom et al. 2014b) showing that vaginal blood engorgement is a relatively slow physiological response. For FIR, SIR and TIR, change scores were calculated for each CS presentation by subtracting mean genital resting baseline from genital response following CS presentation. For genital responses and subjective ratings, effects were tested with repeated measures univariate analysis of variance procedures (general linear model in SPSS), with stimulus and trial as within-subject factors and condition as between-subject factor. The Greenhouse–Geisser correction was applied to adjust for violation of the sphericity assumption in testing repeated measures effects. The preconditioning, acquisition and extinction phases were analyzed separately. Effect sizes are reported as proportion of partial variance (*η*_*p*_^2^) (Cohen 1988). In addition, the strength of the unconditioned and conditioned genital response was determined. The magnitude of the UR was determined by calculating the percentage of preconditioning VPA score (mean VPA in response to the CS+ plus vibration during the acquisition phase / mean VPA in response to the CS+ during the preconditioning phase × 100). The magnitude of the CR was determined by calculating the percentage of the mean VPA in response to vibration (VPA in response to the CS+ during the first extinction trial / mean VPA in response to the CS+ plus vibration during the acquisition phase × 100).

## Results

The participants in the two conditions appeared to differ in age and in prior experience with genital vibrostimulation, see Table 1*Participant characteristics*. Genital data from one participant (from the haloperidol condition) were discarded as outlier since measures from this participant were under 3 SD from the mean (although inclusion of this participant did not change results). There was no relation between the medication the participants had received and the percentage that correctly guessed what they had received (Pearson chi-squared = 2.75, *p* = 0.25), suggesting that blinding was successful. Most participants reported no side effects (*n* = 37). Among the 21 participants who did report side effects, the most commonly reported ones were fatigue, sleepiness and dizziness. Participants in the haloperidol condition reported more side effects as compared to participants in the placebo condition (Fisher’s exact test = 6.05, *p* = 0.03). The most frequent reported side effect was dizziness.

**Table 1:** Participant characteristics

		Placebo (*n* = 29)		Haloperidol (*n* = 29)		
		M	SD	M	SD	*p*
Age (years)		22.24	3.14	20.31	1.71	<0.01*
Sexual functioning (FSFI score)		28.63	5.97	28.81	5.63	0.90
Prior experience vibrostimulation		3.00	1.28	2.24	1.12	0.02*
Pleasantness US		2.24	0.64	2.28	0.65	0.84
US perceived as sexually arousing		2.62	0.78	2.48	0.63	0.46
Declared sexual arousal		2.03	0.73	2.14	0.69	0.58
		Frequency		Frequency		
Use of contraceptives	No use or non-hormonal	5		3		0.72
	Hormonal	24		26		

Descriptive subject variables for each condition. Women’s sexual functioning was assessed by the Female Sexual Function Index (FSFI; Rosen et al. 2000; Ter Kuile et al. 2006). Questions from debriefing, scales: prior experience vibrostimulation 1 (never)–5 (very often); pleasantness US 1 (not pleasant at all)–5 (very pleasant); US perceived as sexually arousing 1 (not sexually arousing at all)–5 (very sexually arousing); declared sexual arousal 1 (not sexually aroused at all)–5 (very sexually aroused)

*p < 0.05

### Genital sexual arousal

#### Preconditioning phase

**Fig. 2:**
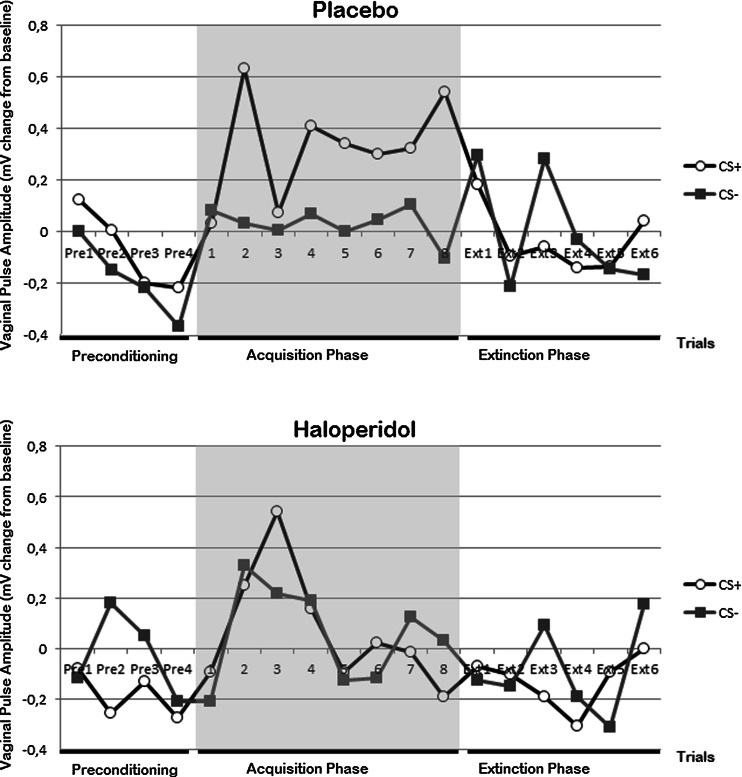
Mean vaginal pulse amplitude (VPA) change scores (with standard error bars) for the conditions placebo and haloperidol, during the third interval response window (TIR) following CS+ and CS− during the preconditioning phase, acquisition phase and extinction phase. Note that during the acquisition phase, the response represents responding to the CS+ plus the US (given that animal studies on conditioned sexual response have revealed interactions between sex steroids and DA in the control of sexual behaviour (see Brom et al. 2014a) and have revealed influences of estrous cycle phase on conditioning and extinction (Milad et al. 2006); additional analyses were conducted, controlled for women during their early follicular phase (i.e. early cycle) and during the late follicular phase (i.e. midcycle). However, those analyses revealed no additional differences between conditions

Analyses were conducted to verify equal levels of VPA in response to the CS+ and CS− during the preconditioning phase. For all latency windows (FIR, SIR and TIR), no difference in VPA following presentation of the CS+ and CS− was found, all *p*s > 0.23. On TIR, a significant stimulus × condition interaction effect was found, *F*(1, 55) = 6.74, *p* = 0.01, *η*_*p*_^2^=.11. As can be seen in Fig. 2, participants in the haloperidol condition demonstrated higher VPA responses towards the CS−, whereas women in the placebo condition had higher VPA in response to the CS+ in the preconditioning phase.

#### Acquisition phase

VPA in response to the vibrostimulation during the acquisition phase was determined in order to verify whether the vibrostimulation served as a sexually arousing US. Genital responses in the second and third latency windows (SIR, TIR) were considered as unconditioned responses. Figure 2 summarizes VPA TIR to CS+ and CS− across trials for both conditions separately. In line with previous studies (Both et al. 2008, 2011; Brom et al. 2014b), the 2 (stimulus) × 8 (trial) × 2 (condition) mixed factors ANOVA of VPA revealed only a significant main effect of stimulus on TIR, FIR *p* = 0.28, SIR, *p* = 0.11, TIR, *F*(1, 54) = 5.65, *p* = 0.02, *η*_*p*_^2^=.10, meaning the CS+ plus vibrostimulation elicited higher levels of VPA. On FIR and SIR, there were no significant stimulus × condition interaction effects, FIR *p* = 0.51, SIR *p* = 0.38, or stimulus × trial × condition interaction effects, FIR *p* = 0.86; SIR *p* = 0.29. On TIR, there was a trend for a stimulus × condition interaction, *F*(1, 54) = 3.16, *p* = 0.08, *η*_*p*_^2^=.06, and for a stimulus × trial × condition interaction, *F*(5, 250) = 2.19, *p* = 0.06, *η*_*p*_^2^=.04. Inspection of Fig. 2 suggests that the placebo condition demonstrated greater differential responding in the second half of the acquisition phase compared to the haloperidol condition. Additional analysis of the first half of the acquisition trials (trials 1–4 of acquisition phase) yielded a significant stimulus × trial × condition interaction effect on TIR *F*(3, 143) = 3.87, *p* < 0.02, *η*_*p*_^2^=.07, and analysis of the second half of the acquisition phase (trials 5–8) yielded a significant stimulus × condition interaction on TIR, *F*(1, 55) = 4.65, *p* < 0.04, *η*_*p*_^2^=.08. Because conditions differed in age, with the placebo condition being significantly older than the haloperidol condition (see Table 1), and in prior experience with vibrostimulation and in the difference in VPA towards CS+/CS− during the preconditioning phase, additional analyses were conducted with those variables as covariates. On TIR, again a significant main effect of stimulus was seen, *F*(1, 51) = 5.23, *p* < 0.03, *η*_*p*_^2^=.10, and a significant stimulus × condition interaction effect, *F*(1, 51) = 6.32, *p* < 0.02, *η*_*p*_^2^=.11. Also, a trend for a stimulus × age interaction, *p* < 0.09, was seen. Concluding, the vibrostimulation resulted in a genital arousal response in both conditions, and results from the additional analysis showed that administration of a DA antagonist decreased the magnitude of differential responding towards the CS+ plus vibrostimulation and CS− in the second half of the acquisition phase.

#### Extinction phase

Analysis of the first extinction trial revealed no conditioned responding, FIR *p* = 0.47, SIR *p* = 0.32, TIR *p* = 0.64, with no differences therein between the conditions, FIR *p* = 0.93; SIR *p* = 0.99; TIR *p* = 0.65. The 2 (stimulus) × 6 (trial) × 2 (condition) mixed factors ANOVA of all extinction trials also did not reveal conditioned responding, all *p*s > 0.19, and no stimulus × trial × condition interaction effect, all *p*s > 0.53. However, a less stringent method, namely analysis of only the response towards the CS+ on the last preconditioning trial and on the first extinction trial, revealed a difference in conditioned responding between the placebo and haloperidol condition, as reflected by a significant trial × condition interaction effect on SIR, *F*(1, 54) = 4.89, *p* < 0.03, *η*_*p*_^2^=.08, and a trend for a trial × condition interaction on TIR *F*(1, 54) = 3.81, *p* < 0.06, *η*_*p*_^2^=.07. Additional analyses were conducted with the variables age, prior experience vibrostimulation and difference in VPA towards CS+/CS− during the preconditioning phase as covariates. Analysis of the first extinction trial again revealed no conditioned responding on FIR and SIR and no differences therein between conditions, all *p*s > 0.28. On TIR, a trend of stimulus was seen, *F*(1, 49) = 3.51, *p* < 0.07, *η*_*p*_^2^=.07, but a non-significant stimulus × condition interaction effect, *p* = 0.11. Additionally, the 2 (stimulus) × 6 (trial) × 2 (condition) mixed factors ANOVA of all extinction trials now revealed conditioned responding on SIR, *F*(1, 49) = 5.25, *p* < 0.03, *η*_*p*_^2^=.10, but no differences therein between conditions, *p* = 0.23. On TIR, no conditioned responding was seen, again with no differences therein between conditions, all *p*s > 0.10. The analysis of only responses towards the CS+ on the last preconditioning trial and on the first extinction trial revealed no difference in conditioned responding between the conditions, all *p*s > 0.10. To conclude, administration of haloperidol did not decrease the magnitude of the conditioned sexual response compared to the placebo condition.

### Subjective measures

#### Preconditioning phase

The 2 (stimulus) × 4 (trial) × 2 (condition) mixed factors ANOVA to verify equal levels of responding to the CSs revealed no difference in responding following presentation of the CS+ and CS− on affective value and subjective sexual arousal, or between conditions, all *p*s > 0.25.

### Extinction phase

#### Subjective affect

**Fig. 3:**
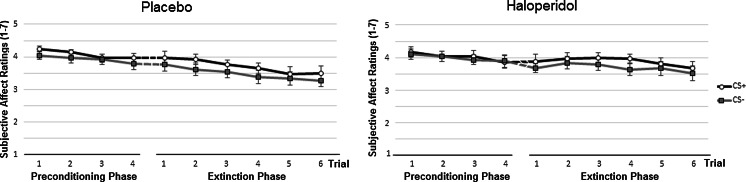
Subjective affect ratings (with standard error bars) following the CS+ and CS− during the preconditioning phase and extinction phase in the two conditions placebo (*left*) and haloperidol (*right*)

As can be seen in Fig. 3, contrary to the expectations, for subjective affect, there was no robust increase of differential responding towards CS+ and CS− after the acquisition phase. Analysis of the first extinction trial revealed no significant stimulus × condition interaction effect, *p* = 0.98, and no main effect of stimulus, *p* = 0.14. Analysis of ratings of affective value during the preconditioning phase (mean trials 1–4) and the first extinction trial revealed no stimulus × condition interaction effect, *p* = 0.83, nor a stimulus × trial × condition interaction effect, *p* = 0.70. The 2 (stimulus) × 6 (trial) × 2 (condition) mixed factors ANOVA of all extinction trials revealed no differences between conditions, as reflected by non-significant stimulus × condition and stimulus × trial × condition interaction effects, all *p*s > 0.64. A trend of stimulus was seen, *F*(1, 48) = 2.97, *p* = 0.09, *η*_*p*_^2^=.06. Analysis with age and prior experience vibrostimulation as covariates showed a similar pattern of results.

#### Subjective sexual arousal

**Fig. 4:**
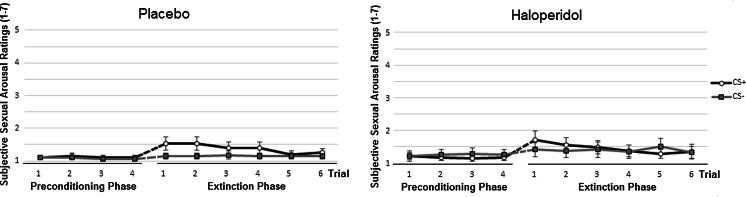
Ratings (with standard error bars) of subjective sexual arousal following the CS+ and CS− during the preconditioning phase and extinction phase in the two conditions placebo (*left*) and haloperidol (*right*)

Figure 4 shows increased ratings of subjective sexual arousal towards the CS+ on the first trials of the extinction phase, indicating conditioned response, in both conditions. Analysis of ratings of subjective sexual arousal during the preconditioning phase (mean trials 1–4) and the first extinction trial revealed no significant stimulus × condition interaction effect, *p* = 0.48, or stimulus × trial × condition interaction effect, *p* = 0.91. However, a significant main effect of stimulus was seen, *F*(1, 54) = 9.71, *p* < 0.01, *η*_*p*_^2^=.15, indicating conditioned responding. Analysis of the first extinction trial yielded no significant stimulus × condition interaction, *p* = 0.76. Again, a main effect of stimulus was found, *F*(1, 55) = 7.06, *p* = 0.01, *η*_*p*_^2^=.11. The 2 (stimulus) × 6 (trial) × 2 (condition) mixed factors ANOVA of all extinction trials revealed no significant interaction effects of stimulus × condition or stimulus × trial × condition, both *p*s > 0.39. However, there was a significant stimulus × trial interaction effect, *F*(3, 148) = 2.76, *p* = 0.04, *η*_*p*_^2^=.06, indicating extinction of conditioned responding. Analyses with age and prior experience vibrostimulation as covariates showed a similar pattern as reported above. To conclude, the modulation of dopaminergic tone with haloperidol did not decrease the magnitude of conditioned subjective affect or sexual arousal as compared to the placebo condition.

### Magnitude of the conditioned genital response

Compared to the unconditioned genital response, the magnitude of the CR during the first extinction trial was 116.8 and 98 %, respectively, for VPA SIR and TIR for the placebo condition. For the DA condition, this was 72.2 and 51.1 %. No significant differences were seen between conditions therein, all *p*s > 0.28.

## Conclusions

To investigate whether DA signalling is a prerequisite in sexual reward learning in humans, dopaminergic tone in healthy women during a sexual conditioning paradigm was manipulated. First, results demonstrated that DA receptor antagonism reduced sexual stimulation-induced genital sexual arousal, emphasizing the importance of DA in unconditional responding to sexual stimulation. However, contrary to the expectations, no differences in conditioned genital responding were seen between the placebo and haloperidol condition after the acquisition phase. Both conditions demonstrated only slight conditioned genital response, but only after correcting for age, prior experience with vibrostimulation and for the difference in genital response towards the CSs during the preconditioning phase. Regarding ratings of affective value, contrary to the expectations, no differential responding towards CS+ and CS− after the acquisition phase could be detected, and contrary to the hypothesis, no differences therein were seen between conditions. For ratings of subjective sexual arousal, women in both the placebo and haloperidol condition demonstrated increased ratings towards the CS+ on the first trials of the extinction phase. However, the conditions did not differ in this conditioned response.

Results from the present study suggest that DA availability indeed contributes to unconditioned behavioural responses to sexual rewarding stimuli. This is in accordance with previous work that showed that DA systems are involved in (sexual) reward signalling (Both et al. 2005; Brom et al. 2014a; Georgiadis and Kringelbach 2012; Oei et al. 2012). Quite intriguing is the finding that DA downregulation did not seem to affect subsequent conditioned genital response and conditioned subjective sexual arousal. However, it is important to keep in mind that only very weak conditioned genital responding was seen, making it not straightforward to conclude that administration of the DA receptor antagonist haloperidol did not influence conditioned sexual response in healthy women. This is also evidenced by the data on the magnitude of the genital conditioned response. This finding of only mild conditioned genital response is surprising, especially when considering that similar parameters to those of previous research were used, when evidence for genital conditioning effects were found (Both et al. 2008, 2011; Brom et al. 2014b, c). Compared to this previous research, in the present study, women in both conditions rated the US as less pleasant and less sexually arousing. Although we do not have a clear explanation for why women experienced the US as less sexually arousing and why no conditioned genital response could be detected in the placebo condition, we should mention that sexually conditioned genital responses have generally been found to be small (Brom et al. 2014a; Hoffmann et al. 2004; O’Donohue and Plaud 1994). Of importance, women in the placebo condition had significantly more experience with genital vibrostimulation. Since the attribution of incentive salience is the product of previous experience (i.e. learned associations; habituation) interacting with someone’s genetic propensity and neurobiological state (Flagel et al. 2011), it could be that the US was less effective and rewarding for participants in the placebo condition. The above makes clear that further replications of the sexual conditioning results in independent samples are highly important.

Although administration of haloperidol resulted in an attenuated unconditioned genital response, this did not seem to affect the perceived pleasantness or sexual arousability of the US or the magnitude of conditioned subjective sexual arousal. Moreover, in an earlier study on sexual response (Both et al. 2005), levodopa seemed only to increase T reflex magnitude in response to sexual stimulation in men (and not in women), whereas genital and subjective sexual arousal were not affected by levodopa. This suggests that subjectively reported feelings may not be affected by DA signalling. The conscious awareness of a motivational state may be dissociable from the underlying motivational processes (Berridge 1996; Berridge and Kringelbach 2008). Moreover, the fact that sexual response systems can diverge has long been recognized, especially in women (Chivers et al. 2010; Laan and Everaerd 1995; Laan et al. 1995). Women can show increases in VPA, while no increases in self-reported sexual arousal are observed, or vice versa.

Several limitations of the current study should be noted. Although we may assume that the administration of 3 mg haloperidol 3 h before the start of the experimental procedure indeed effectively inhibited dopaminergic tone (Darby et al. 1995; Liem-Moolenaar et al. 2010; Nordstrom et al. 1992; Oei et al. 2012), also reflected by the difference in reported adverse effects between the haloperidol and placebo conditions, future studies should incorporate additional sensitive measurements of drug-induced CNS effects (Liem-Moolenaar et al. 2010), to assure testing during maximal plasma concentrations. Moreover, since haloperidol exhibits polypharmacology (i.e. it may affect multiple receptor proteins in the nervous system; Seeman 2002; Videbaeck et al. 2001), future studies on sexual reward learning in humans should preferably make use of positron emission tomography and selective ligands in order to be able to attribute its effects to action on D2 receptors. Second, the present study sample exclusively comprised women. Results from a fMRI study by Klucken et al. (2009) on sexual conditioning revealed stronger conditioned activation in the amygdala, thalamus and occipital cortex in men compared to women. The researchers considered these results to be in line with other findings (Gutiérrez and Domjan 1997; Pfaus et al. 2001). Research has demonstrated that gender differences in the number of DA neurons are influenced by several factors, including sex chromosome complement (Lombardo et al. 2012), the presence of the SRY gene (Dewing et al. 2006) and gonadal hormones. Moreover, it is suggested that testosterone regulates incentive sensitivity through interactions with mesolimbic DA pathways (Hermans et al. 2010; Wood 2008). Additionally, previous studies have reported conflicting results about the effects of DA on female sexual motivation in animals and humans (Both et al. 2005). This and present findings make clear that future research on the role of DA in sexual learning in both sexes in humans is warranted, as these findings may help in the understanding of the biological mechanisms underpinning addictive behaviours and how these may affect vulnerability to drug abuse or the development of sexual dysfunctions in men and women. In the present study, almost all participants used hormonal contraception. It is known that hormonal contraception may have an influence on the neurochemical regulation of dopaminergic midbrain areas involved in neurobiological processes, herewith affecting reward learning (Brom et al. 2014a; Pletzer and Kerschbaum 2014; Sotomayor-Zarate et al. 2014). Therefore, future studies on sexual reward learning in women should preferably include a larger sample of women, in order to investigate the influence of hormonal contraceptives on sexual reward learning. Additionally, in the present study, vaginal photoplethysmography was used as indicator of physiological sexual arousal. Vaginal engorgement, however, is only one of many co-occurring processes during the sexual arousal response. Functional imaging studies on the role of DA in sexual reward learning in healthy men and women may provide complementary insight in neurochemical mechanisms involved in sexual behaviours, which may help foster potentially critical insights in the aetiology of disorders in sexual motivation. Since sexual arousal can eventually result in overt behaviour such as approach and consumption (Dekker and Everaerd 1989), future studies should also incorporate a behavioural task to assess automatic action tendencies (Brom et al. 2014b; Wiers et al. 2011). Lastly, another limitation of the present study is the absence of a between-subjects (unpaired) control group. Such a control group would help to distinguish learning about the CS+ and the CS− (Domjan 2010; Hoffmann et al. 2014). Therefore, making use of such a control group in future research is desirable.

In conclusion, the current study is the first that investigated the role of DA in human sexual reward learning. The present results do not indicate an effect of DA antagonism on conditioned sexual response in women. However, effects of inhibiting dopaminergic tone with a DA antagonist (haloperidol) were seen in the magnitude of unconditional genital responding to sexual stimulation. Future studies on the role of DA in human sexual reward learning are warranted, while facilitation as well as impairment of sexual reward learning is relevant in the context of treatment of hyposexual and hypersexual desire disorder.
